# A Nursing Care Model for Surge Capacity Management in Intensive Care Units During the COVID-19 Pandemic: Experience From Qatif Central Hospital, Saudi Arabia

**DOI:** 10.7759/cureus.48193

**Published:** 2023-11-02

**Authors:** Mohammed I Al Bazroun, Alhasan Almahrouq

**Affiliations:** 1 Nursing Professional Development, Qatif Health Network, Qatif, SAU; 2 Nursing Affairs, Qatif Health Network, Qatif, SAU

**Keywords:** nursing care model, surge plan, nursing, critical care, covid-19 pandemic

## Abstract

Introduction

The COVID-19 pandemic resulted in a surge of critically ill patients requiring intensive care. This posed challenges for healthcare systems in managing increased ICU bed demands with limited resources.

Methods

A retrospective qualitative review of institutional documents and plans was conducted. Key strategies related to ICU bed expansion, nursing staff classification and training, clinical supervision, and performance evaluation were analyzed.

Results

Qatif Central Hospital increased ICU beds from 20 to 50 by converting other clinical areas. Nursing staff were categorized based on critical care experience, and additional training was provided to non-ICU nurses. A preceptor-led nursing care model with staff responsibilities was implemented. Periodic evaluations ensured continued competence.

Conclusion

The nursing care model at Qatif Central Hospital effectively facilitated ICU surge capacity while maintaining care quality. The model offers a viable framework for other healthcare institutions facing similar challenges. However, the study is limited by its retrospective design and focus on a single institution.

## Introduction

The coronavirus pandemic, caused by severe acute respiratory syndrome coronavirus 2 (SARS-CoV-2), resulted in an unprecedented burden on global healthcare systems, particularly intensive care units (ICUs) [1,2]. Initially emerging in Wuhan, China, in late 2019, the virus quickly led to severe complications in a subset of COVID-19 patients, such as acute respiratory distress syndrome (ARDS) and multi-organ system dysfunction requiring intensive interventions [3-6].

Early hotspots like Hubei province in China and Lombardy in Italy exceeded their baseline ICU capacities [7,8]. An abrupt spike in demand for isolation rooms, ventilators, and specialized staff led healthcare systems to the brink of collapse [9,10]. In response, makeshift field hospitals were constructed, and medical personnel faced difficult resource allocation decisions, often without adequate personal protective equipment [5-7].

To prepare for an impending crisis, modeling studies warned of dire outcomes in the United States and Europe if immediate action was not taken to increase ICU capacities [8]. Despite efforts, subsequent waves of infection placed a persistent strain on ICUs worldwide [10].

States such as New York and Massachusetts were particularly hard hit, with demands for ventilators and ICU beds tripling in some areas [9,11]. Shortages of specialized nurses trained to manage complex cases compromised both the quality of care and clinical outcomes for COVID-19 and non-COVID patients [12]. At the population level, the demand for critical care in the United States far exceeded capacity, necessitating robust containment and preparation strategies [12].

The term “surge” refers to the ability to temporarily bolster healthcare infrastructure to meet spikes in demand [13,14]. Enhancing ICU surge capacity thus became an urgent public health imperative [13]. Achieving this required overcoming significant logistical, educational, and safety challenges [15].

Nurses serve as the backbone of ICU care. The effective deployment and training of this essential workforce became vital for managing ICU capacities. Consequently, transforming nursing practice standards became a high stake endeavor crucial for the global surge response.

This retrospective qualitative review aims to provide a comprehensive synthesis of evidence on nursing care models that enhanced ICU surge capacities during the acute phases of the COVID-19 crisis. Identifying navigated barriers and lessons learned could inform future preparedness, particularly concerning scalable nursing workforce structures, skill-mix strategies, safety protocols, and clinical standardization. This is essential for maintaining high-quality critical care access during public health emergencies.

## Materials and methods

The retrospective qualitative review was conducted at Qatif Central Hospital and focused on institutional documents and records spanning March to June 2020. During this period, the hospital implemented strategies to manage a surge in ICU patient admissions due to the COVID-19 pandemic. Data were collected from multiple sources, including hospital administrative databases, electronic health records, training logs, and policy documents. The key components analyzed were the key areas essential to managing ICU surge capacity. The approach to expanding ICU bed numbers was examined in detail, focusing on physical arrangements and resource allocation. Concurrently, the methods and criteria for categorizing nursing staff were evaluated, drawing upon human resource documents and duty rosters for analysis. Training modules, curricula, and assessment criteria were scrutinized to understand how the nursing workforce was rapidly upskilling, encompassing both formal training sessions and on-the-job experiences. Additionally, the care delivery model adopted was subjected to a thorough analysis, taking into account role distributions and staffing ratios, and assessed using institutional policies and guidelines. Lastly, an in-depth review was conducted on how responsibilities were delineated among staff members, as well as the tools and metrics employed for periodic competency evaluations.

Study setting

The review was conducted at Qatif Central Hospital, a secondary healthcare institution well-equipped with ICU facilities.

Data collection

Two independent reviewers extracted relevant data from these sources. A standardized format was employed for data compilation to facilitate uniform analysis. In cases where discrepancies emerged in data extraction, these were resolved through consensus-based discussions among the review team.

Ethical considerations

This review utilized routinely collected programmatic data and did not involve direct contact with patients. Approval from the Qatif Central Hospital ethical board was obtained before conducting the analysis.

## Results

**Figure 1:**
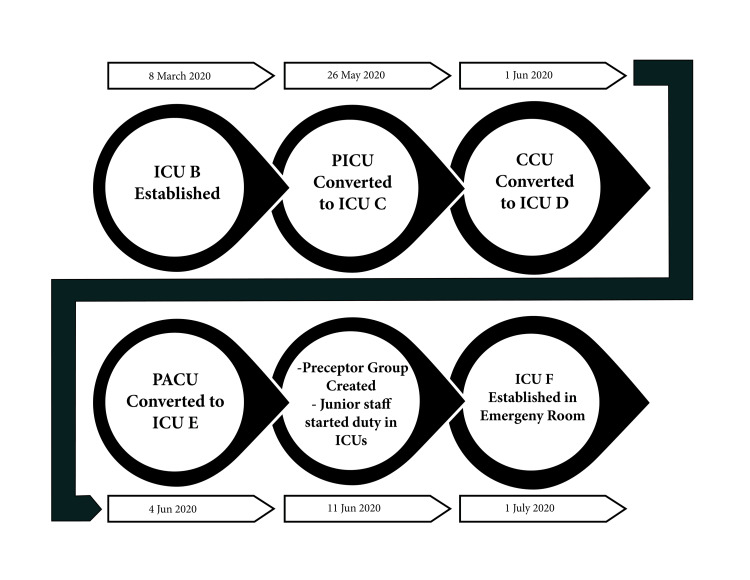
Summary of the surge plan process

ICU bed capacity was increased from 20 to 50 beds (Table 1) through conversions of other clinical areas like the post-anesthesia care unit, coronary care unit, and emergency department spaces (Figure 1). This 30-bed surge maintained ICU occupancy rates below 80% throughout the initial pandemic period, ensuring adequate critical care capacity for all patients requiring intubation and mechanical ventilation. 

**Table 1:** ICU bed capacity and staff numbers

Department Name	Total Beds Capacity	Total of Nursing Staffs Number
ICU A	20	66
ICU B	7	20
ICU C	8	28
ICU D	6	24
ICU E	5	24
ICU F	4	20
Total	50 beds	182 staffs

The tiered nursing workforce model supported the deployment of 70 nurses in total. This included 20 ICU nurses designated as preceptors, 30 nurses with other critical care experience, and 20 rapidly trained through the COVID-19 course. This expanded nursing staff pool allowed optimal nurse-to-patient ratios of 1:1-2 to be maintained as the census fluctuated.

Nursing workforce classification and training

Nursing staff were categorized as those with 1) adult ICU experience, 2) experience in other critical care areas, 3) procedural areas experience, and 4) non-ICU nurses. Non-ICU nurses underwent a COVID-19 critical care training course plus clinical orientation.

To equip the nursing staff with the skills required for effective ICU surge management, a comprehensive training program was done. Lasting for two weeks, the program was divided into modules covering key aspects of critical care nursing. These included mechanical ventilation management, hemodynamic monitoring, and strict infection control procedures. A blended learning approach was employed, combining traditional classroom instruction with hands-on simulation exercises. The program also included a series of assessments consisting of written examinations, practical skill tests, and a final evaluation by senior nursing staff to ensure competency in critical care procedures.

Nursing care model 

**Figure 2:**
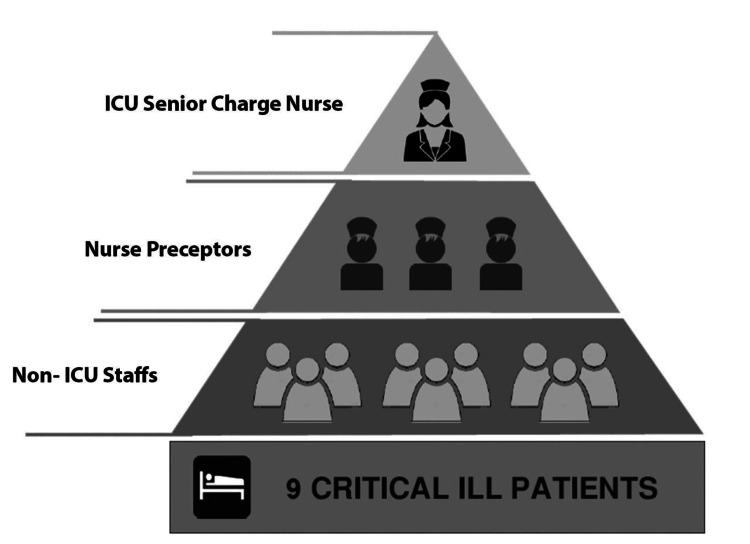
Nursing care model

A preceptor-led model was implemented. Experienced ICU nurses managed nurse-to-patient ratios of 1:1-2, while non-ICU nurses managed 1:1 nurse-to-patient ratios under ICU preceptor supervision. Preceptors guided, supervised, and evaluated primary nurses (Figure 2). 

Performance evaluation

Non-ICU nurse competencies were periodically evaluated using checklists across skills and knowledge. Feedback was obtained from unit heads, preceptors, and clinical instructors to identify further training needs.

## Discussion

The Qatif Central Hospital strategy enabled the rapid expansion of ICU facilities by 30 beds through prudent infrastructure utilization. The nursing care model facilitated the phased deployment of the available nursing workforce based on their capabilities. The guidance of experienced preceptors supported non-ICU nurses in clinical adaptation. Periodic evaluation strengthens critical care competencies through continuous feedback and learning. This holistic approach ensured continued high-quality care despite the surge in critically ill patients during the COVID-19 pandemic.

The Qatif Central Hospital nursing care model aligns with several recommendations from the literature on ICU surge capacity management during mass casualty infectious disease events. Similar to strategies proposed by Christian et al. (2014) and Sprung et al. (2010), Qatif Central Hospital expanded ICU infrastructure by rapidly converting other clinical areas into temporary ICU beds. This approach to prudent infrastructure utilization optimized available space, as suggested [16,17].

The nursing workforce classification adopted at Qatif Central Hospital was informed by evidence that supporting surge operations requires mobilizing additional clinicians through rapid training programs [18].This is consistent with recommendations by Devareaux et al. (2008) and guidelines on optimizing staffing in times of crisis discussed by Chung and Sohn (2018) and Aragon Penoyer (2010) [19,20]. The tiered classification ensured nurses were deployed commensurate with their skill levels.

Implementation of a preceptor-led nursing care model mirrors frameworks suggested in the literature [12,17]. Maves et al. (2020) and Sprung et al. (2010) emphasize the importance of guidance by experienced staff to facilitate urgent orientation of additional personnel. The model effectively supported capacity expansion while upholding quality of care in line with optimal staffing guidelines. 

Limitations 

The study presents several limitations that warrant discussion. First, its single-center focus limits the generalizability of the findings. This limitation is particularly crucial given the variability in healthcare infrastructure, staff resources, and patient populations across different settings. Second, the retrospective nature of the study introduces potential biases. Data quality could be compromised due to inaccuracies in institutional records, especially those hastily documented during crisis periods. Third, the study did not evaluate long-term outcomes, which raises questions about the sustainability and efficacy of the implemented strategies beyond the immediate crisis.

Recommendations

The review yields several recommendations for healthcare systems facing similar surge challenges. First, detailed surge capacity plans must be developed to rapidly expand ICU infrastructure and mobilize additional clinical staff. A tiered nursing workforce model is suggested for efficient clinician deployment based on their critical care experience. Intensive onboarding and preceptorship programs are essential for rapidly acclimating new staff to ICU procedures. Moreover, the establishment of regional healthcare coalitions could facilitate staff sharing and mutual aid, thereby alleviating strain on individual facilities. The use of tele-ICU programs is also recommended for managing lower-acuity patients remotely, thereby freeing up essential resources. Additionally, crisis standards of care policies should be enacted to permit flexibility in care delivery models, such as altering nurse-to-patient ratios while maintaining quality. Finally, sustained investment in simulated surge response exercises and crisis management tools is crucial for long-term preparedness.

## Conclusions

This study delineates a targeted approach to ICU surge capacity management, focusing on nursing care, as demonstrated by Qatif Central Hospital during the COVID-19 pandemic. The implemented model stands as a practical example of how strategic human resource management, specialized training, and ongoing competency assessments can collectively address the challenges posed by sudden increases in ICU demand. The findings substantiate that a well-planned nursing model not only optimizes resource allocation but also maintains high care quality standards. However, it is crucial to recognize that the success of any ICU surge plan is contingent upon its integration into a broader, multi-disciplinary crisis management strategy. While this study provides valuable insights into the role of nursing care in ICU surge management, further research is needed to assess its impact on patient outcomes and validate its efficacy in diverse healthcare settings.

To fortify healthcare systems against future pandemics or mass casualty events, a sustained commitment is essential, both in terms of investment in healthcare infrastructure and in the regular updating and reviewing of surge capacity plans and clinical guidelines.
